# Histological, Genotoxic, and Biochemical Effects on* Cnesterodon decemmaculatus* (Jenyns 1842) (Cyprinodontiformes, Poeciliidae): Early Response Bioassays to Assess the Impact of Receiving Waters

**DOI:** 10.1155/2019/4687685

**Published:** 2019-01-01

**Authors:** Natalia Alejandra Ossana, Federico Gastón Baudou, Patricia Mónica Castañé, Luis Tripoli, Sonia Soloneski, Lucrecia Ferrari

**Affiliations:** ^1^Programa de Ecofisiología Aplicada (PRODEA), Instituto de Ecología y Desarrollo Sustentable (INEDES, CONICET-UNLU), Universidad Nacional de Luján (B6700ZBA), Luján, Argentina; ^2^Consejo Nacional de Investigaciones Científicas y Técnicas (CONICET), Godoy Cruz 2290, (C1425FQB), Buenos Aires, Argentina; ^3^Cátedra de Citología, Facultad de Ciencias Naturales y Museo, Universidad Nacional de La Plata, Calle 64 N° 3, B1904AMA, La Plata, Argentina

## Abstract

In the present study, the toxicity of receiving waters from a highly polluted urban watercourse, the Reconquista River, Argentina, collected at a dam in the upstream part of the river was evaluated.* Cnesterodon decemmaculatus, *a widely distributed fish species in Pampasic rivers proposed for use in ecotoxicological evaluations, was used as a test organism. A 96-h acute toxicity bioassay with river water quality which has been characterized as moderately contaminated was performed. The treatment groups were (1) whole surface river water; (2) whole surface river water with 2 mg Cd/L added as a simulated metal contaminant pulse; (3) a negative control using reconstituted moderately hard water (MHW); (4) a metal positive control, MHW + 2 mg Cd/L; and (5) a positive genotoxicity control, MHW + 5 mg Cyclophosphamide/L (CP). The condition factor rate, micronuclei frequency, and comet assay from peripherical blood, structural changes of the gill arrangement by scanning electron microscope (SEM) analysis, histopathological changes in the liver and the glutathione-S-transferase, catalase, superoxide dismutase, glutathione, and protein content from the body midsection (viscera) were evaluated. According to our results, for short term exposure, SEM analyses of gills and liver histopathological analyses could be useful tools for the evaluation of target organ damage as well as comet assays for DNA damage. We propose that the 96-h laboratory bioassay protocol described is useful for monitoring the deterioration of water quality employing the teleost* C. decemmaculatus* and that the microscope analysis of gills and liver as well as the comet assay methodology could be sensitive endpoint indicators.

## 1. Introduction

The Reconquista River, which is part of the great del Plata Basin, is a lowland watercourse in the Pampasic region located in the north-east part of Buenos Aires province, Argentina. It runs in a SW to NE direction, and in its route of approximately 50 km it receives contributions from small water courses [1]. This river is highly polluted with industrial and domestic outputs from point and diffuse sources. Rigacci et al. [2], who studied the effect of a reservoir (Roggero dam) located at the headwaters of the Reconquista River, observed an increased water quality deterioration rate. There is a considerable decrease in biodiversity along the Reconquista River, where the biota is affected by a chronically disturbed environment with pollution hotspots and marked water-level fluctuations [3]. One of a few fish species usually reported in the river up to its middle section is the ten-spotted live-bearer* Cnesterodon decemmaculatus* (Jenyns 1842) (Cyprinodontiformes, Poeciliidae). This species can be found in pristine as well as in severely degraded habitats [4], and it has been successfully used as a bioindicator to evaluate the toxicity of pesticides [5–7], metals, and receiving waters [3, 8–12]. At the regional level, the number of studies in order to characterize* C. decemmaculatus* as a neotropical test species for ecotoxicological evaluations has increased in last years.

Cadmium (Cd) is a nonferrous metal with unknown physiological function. It can replace essential metals such as copper and zinc in several metalloproteins, altering the protein conformation and affecting their activity, because this element interacts ubiquitously with sulfhydryl groups of amino acids, proteins, and enzymes [13]. Thus, the toxic effects of Cd are related to changes in the natural physiological and biochemical processes in organisms, and it has been proposed as a reference toxicant for* C. decemmaculatus* in ecotoxicological assays [10, 12]. In water bodies, contamination by several xenobiotics, such as pesticides and metals, is mainly point-specific; therefore, their concentrations in surface waters fluctuate and depend on the physicochemical properties of the molecules and the physicochemical profile of the receptor medium. Cd is rapidly made nonbioavailable, mainly by complexation processes with particulate material and humic substances present in natural waters; for a short time it can be at lethal levels for the biota, and in consequence the homeostatic capacity of individuals would become responsible for dampening or not its effect [14]. Cd is sometimes found at levels higher than those established by guidelines for the protection of aquatic biota [3, 9].

It is well known that environmental contaminants can alter homeostatic parameters in fish and some of these parameters can be used as biomarkers. Thus, biochemical, physiological, histological, morphological, and behavioral measurements used as biomarkers become sensitive tools that can be used to assess the adverse effects of several pollutants or unknown mixtures thereof (like receiving waters) both* in situ* and in laboratory experimental conditions. These biomarkers may be able to provide an early warning signal well before severe environmental degradation has already occurred [15].

The analysis of micronuclei frequency (MN) and the induction of DNA single-strand breaks by the comet assay are the most frequently employed endpoints for detecting DNA damage in circulating peripheral blood erythrocytes in fish [5, 16, 17]. Likewise, histopathological changes in the gills and liver have been proposed as useful tools for monitoring fish health in polluted water bodies [18]. Particularly for fishes, injury changes in the gill epithelium and the hepatic tissue were found to be good pollution indicators, as these are the main target organs for xenobiotics [19, 20]. On the other hand, the liver accumulates many toxic compounds, and it is the primary organ for the biotransformation of organic xenobiotics, excretion of harmful trace metals, food digestion and storage, and metabolism of sex hormones [21, 22].

Although organisms have defenses against reactive oxygen species (ROS) overproduction, host exogenous processes like environmental pollution cause disequilibrium between the excessive ROS formation and the limited antioxidant defenses [23]. ROS are generated during metabolism and by numerous pollutants present in the aquatic environment, like heavy metals, pesticides, hydrocarbons, etc., leading to oxidative stress conditions in organisms. Fish possess well-developed antioxidant defense systems to remove increased ROS. These systems include antioxidant enzymes such as superoxide dismutase (SOD) and catalase (CAT), glutathione-S-transferase (GST), and reduced glutathione (GSH) which is a low molecular weight thiol that can react directly with ROS [24]. The evaluation of the levels and activities of these enzymes and molecules enable the detection of adaptive responses to the formation of ROS triggered by the entrance of toxic substances into the organisms [25].

The aims of this study were (1) to provide information on the response ability of a set of biomarkers in* C. decemmaculatus* under short term exposure to receiving waters from the Reconquista River; (2) to add information concerning the characterization of responses to Cd, such as the toxic reference; and (3) to assess these early-effects biomarkers as endpoints in this neotropical test organism, thus contributing to the validation of its use in biomonitoring acute stress due to contamination. We employed MN analysis and the alkaline comet assay as genotoxic endpoints. Additionally, histopathological changes in the gills and liver were also evaluated along with quantification of the somatic index and antioxidant defenses such as glutathione, catalase, superoxide dismutase, and protein content.

## 2. Materials and Methods

### 2.1. Surface Water Sample from the Reconquista River

Samples of surface water were taken in autumn from the Roggero dam (34°40′16.47′′ S, 58°52′46.19′′ W) located in the boundary area between the upper and middle basins of the Reconquista River. Although built to reduce overflow due to flooding, the reservoir could be considered a depuration system for transported material and, therefore, for water quality. This is particularly significant since water from various streams enters the reservoir, transporting sewage water among other contaminants [2].

For the bioassay, a 20-L water sample was collected in plastic containers, stored at 4–8°C, and used within 48 h after sampling. Temperature, pH, and conductivity were measured* in situ* with an electrochemical portable device (HqD Field case Hach). Surface water samples for physicochemical analyses were conditioned in clean bottles and immediately transported to the laboratory in coolers containing ice and stored at 4–8°C until analysis. Samples for heavy metal determinations were collected in plastic bottles and kept acidified with HNO_3_ (pH ≤ 2), while those for pesticide determinations were collected in amber-colored glass bottles. No rainfall was recorded for 3 days before water sampling.

A water quality index (WQI) for organic pollution was calculated based on the dissolved oxygen, chlorides, biochemical oxygen demand, and ammonium [26]; it is unitless and ranges from 0 (highly polluted) to 10 (high purity).

Screening of organochlorine and organophosphate pesticides was performed on river samples using high-resolution capillary gas chromatography (Hewlett Packard 61530 Plus A6890) after liquid-liquid extraction with dichloromethane [27] and clean-up with Fluorisil [28] with appropriate capture detectors (electron capture, flame photometric, and nitrogen phosphorous) [27]. Screening included the following pesticides: Organochlorines: aldrin, DDT and their metabolites, dieldrin, *α*- and *β*-endosulfan, endrin, heptachlor, heptachlor epoxide, hexachlorobenzene, *α*- and *β*-hexachlorocyclohexane; Organophosphates: chlorfenviphos, chlorpyriphos, coumaphos, diazinon, ethylbromophos, ethion, fentrothion, malathion, and methylparathion. The detection limit was 0.03 *µ*g/L for organochlorines and 0.02 *µ*g/L for organophosphates.

Concentrations of arsenic (As), zinc, (Zn), copper (Cu), chromium (Cr), cadmium (Cd), and lead (Pb) (Methods 3111B, 3113 B, 3114) [27] in the river water sample, all assay media, and the moderate hard water (MHW, controls) were measured using a Perkin Elmer analyst 200 atomic absorption spectrophotometer. Arsenic content was determined by atomic absorption using a FIAS 100 model flow injection module. Cd, Cr, and Pb were measured using a HGA900 model graphite furnace, while Cu and Zn were measured using an air acetylene torch. Metal certified standards were used (1,000 g metal/L, Merck). Results are expressed as the mean value of two or three readings. The detection limit was in the range of 0.5–1.0 *µ*g/L.

### 2.2. Animal Source and Experimental Design

Specimens of* C. decemmaculatus* mostly female were collected from our own outdoor culture [29] and transferred to an indoor culture for a holding period in dechlorinated tap water with controlled temperature and photoperiod (21±1°C; 16 h light/8 h dark). At the beginning of the 15-d acclimation period, fish were randomly distributed in 14x14x20-cm aquaria (ten fish per aquarium, load <1 g/L) which contained reconstituted MHW (pH: 7.4-7.8; hardness: 80-100 mg CaCO_3_/L; alkalinity: 60-70 mg CaCO_3_/L) [30]. This media was chosen because it is similar to river water composition. Aquaria were placed inside a permanently aerated incubation chamber at the same temperature and photoperiod. Fish were fed daily on crushed flake food (TetraFin®). Access to food was* ad libitum* during the holding and acclimation periods and it was restricted to 2% w/w during the bioassay. Fish used in the experiment had a mean ± SEM (standard error media) weight and total length of 113.62 ± 4.98 mg and 25.89 ± 0.33 mm, respectively. Of the total of the animals used, 75-85% were females.

The bioassay was performed in accordance with previously described experimental design [11]. At the beginning of the experiment, fish were exposed in duplicate for 96 h to one of the following treatments: (1) whole surface river water (RR); (2) whole surface river water with 2 mg Cd/L added as a simulated metal contaminant pulse (RR+Cd), a concentration of heavy metals frequently found in our rivers; (3) a negative control using moderately hard water (NC); (4) a metal positive control, MHW + 2 mg Cd/L (Cd); and (5) a positive genotoxicity control, MHW + 5 mg Cyclophosphamide/L (CP). Ten individuals were used for each replicate. Hardness, dissolved oxygen (DO), pH, conductivity, and Cd concentration were monitored daily in each replicate.

### 2.3. Biological Parameters

After the exposure period, animals were anesthetized by placing them in ice water. Then, they were weighed (mg) and their length (mm) was measured to calculate Fulton's condition factor [K = 100 x body weight/(total length)^3^].

Fish were euthanized by incision behind the operculum. This method of euthanasia was chosen in order to avoid factors that may confuse the enzymatic response, and it follows the recommendation of the National Institutes of Health Guidelines [31] (Resolution 672-15, National University of Lujan). A drop of blood was smeared onto precleaned slides for MN detection, and another drop was collected in an Eppendorf microtube for comet assay (see Section 2.3.2). Then, for the determination of biochemical biomarkers, the body midsection (M) of the fish comprising the section behind the operculum up to the anus containing the viscera was used. This methodology was adopted considering the small size of the animals and has been previously used by other authors [6, 32].

At the end of exposure time a subgroup of fish was destined for histology and other subgroup for the remaining biomarkers.

#### 2.3.1. Histological Preparation of the Gills and Body Midsection

Gills were carefully excised and histologically processed. Briefly, the gills were washed repeatedly at room temperature in 1% phosphate buffer 0.1 M (pH 7.2) to remove residuals, fixed with 2.5% glutaraldehyde for 2 h, and finally washed with the same buffer for 48 h. Subsequently, gills were dehydrated in a graded ethanol series (10-30-50-70%) and stored in 70% ethanol until being dried by the critical-point technique, coated with gold-palladium, and mounted on bronze stubs [33]. Gills were examined under a Philips XL series 30 or a Carl Zeiss NTS SUPRA 40 scanning electron microscope.

For hepatic histological evaluation, the body midsection of some individuals was fixed in Bouin for 24 h, dehydrated through a graded series of ethanol, cleared in xylene, and embedded in paraffin. Paraffin cross-sections were cut into 5-*µ*m thick slices and stained with hematoxylin and eosin. Samples were examined under a light microscope (Carl Zeiss Primo Star) and photomicrographs were taken with a digital camera (Canon) using the AxioVision 4.8 software. For assessing hepatic damage, two or three sections per specimen and four randomly selected fields per section were examined. The following histological alterations were chosen to quantify damage: hepatocytes with pyknotic nuclei, melanomacrophage centers, hyperemia, and hemorrhages [34]. A total of 18 to 21 fields were examined per each treatment and the records were contrasted against control.

#### 2.3.2. Micronuclei Frequency and Comet Assay

One drop of peripheral blood from each animal was smeared onto clean slides, then air dried, fixed with 100% (v/v) cold methanol (4°C) for 20 min, and stained with 5% Giemsa solution for 15 min. The micronuclei frequency (MN) was determined by analyzing 1500 mature erythrocytes from each fish as suggested previously [5, 11] and expressed as the total amount of MN per 1000 cells. MN frequency was scored using previously described criteria [35].

For the comet assay, a drop of peripheral blood was collected in an Eppendorf microtube with 1.5 mL phosphate buffered saline (PBS) and centrifuged at 2000 rpm for 8 min at room temperature. The pellet was resuspended in a final volume of 70 *µ*L of 0.5% low-melting-point agarose and layered on a slide precoated with 100 *µ*L of 0.5% normal-melting-point agarose. The slide was covered with a coverslip and placed at 4°C for 12 min, then the coverslip was removed and the slide was covered with a third layer of 50 *µ*L 0.5% low-melting-point agarose. After solidification, the coverslip was removed, and slides were immersed in a lysis solution (2.5 M NaCl, 100 mM Na_2_EDTA, 10 mM Tris, pH 10, 1% Triton X-100 and 10% DMSO) and lysed in darkness for 1 h at 4°C. Then, slides were placed in an electrophoresis buffer (1 mM Na_2_EDTA, 300 mM NaOH) for 15 min at 4°C followed by electrophoresis in the same buffer and temperature for 10 min at 25 V and 250 mA. Finally, the slides were neutralized with a solution comprising 0.4 mM Tris-HCl at pH 7.5 and stained with 4′,6-diamino-2-phenylindole (DAPI; Vectashield mounting medium H1200; Vector Laboratories, Burlingame, CA, USA) immediately before observation under the microscope. The extent of DNA damage was quantified by the length of DNA migration, which was visually determined in 100 randomly selected and nonoverlapping cells. DNA damage was classified into four types (0-I undamaged, II minimum damage, III medium damage, and IV maximum damage). Data were expressed as the percentage of damaged cells (sum of types II-IV). We also calculated the genetic damage index (GDI) using the formula GDI = [(I) + 2*∗*(II) + 3*∗*(III) + 4*∗*(IV)]/N, where N represents the total number of cells scored [36]. Slides were coded and blind-scored under a Carl Zeiss Primo Star microscope and a Carl Zeiss HBO50 epifluorescence microscope at 1000X magnification for the MN and comet assay techniques, respectively.

#### 2.3.3. Biochemical Biomarkers

Enzyme extracts for the body midsections were prepared from individual fish according to Ossana et al. [37]. Briefly, sections were homogenized in phosphate buffer at a pH of 7.4. The samples were centrifuged at 10,000 G and 4°C for 10 min, and the resultant supernatant was used to assess the biochemical determinations. Protein content was determined according to Lowry et al. [38], using bovine serum albumin as a standard. CAT activity was determined using H_2_O_2_ as a substrate at 240 nm following Baudhuin et al. [39]. SOD activity was calculated as the 50% inhibition of cytochrome C reduction by competition with SOD for the superoxide anion radical formed by the xanthine/xanthine oxidase system at 550 nm following the method suggested by McCord and Fridovich [40]. GST was determined using 1-chloro-2,4-dinitrobenzene as substrate at 340 nm, following Habig et al. [41]. Glutathione content (GSH) was determined using trichloroacetic acid, and thiolate anion formation was determined at room temperature at 412 nm following Ellman's method [42]. All measurements were carried out in triplicate and calculations were made on the basis of the average percentage of the normalized values. Enzyme activities were calculated in terms of the sample protein content.

### 2.4. Statistical Analyses

Data on biomarkers are reported as the mean ± SEM. Assumptions of normality and homoscedasticity were tested with the Kolmogorov–Smirnov and Bartlett tests, respectively [43]. Statistical comparisons between treatments and control values were analyzed using the parametric analysis of variance (ANOVA) followed by Tukey's multiple comparison test or the nonparametric Kruskal–Wallis test followed by Dunn's multiple comparison test [43]. The significance level was set at p < 0.05. Data were statistically analyzed with the InfoStat program [44].

## 3. Results

### 3.1. Physicochemical Profile of River Water Sampled and the Media of the Bioassays

Table 1 shows the values of the physicochemical parameters measured and the WQI calculated from the surface water sample of the Reconquista River. The recorded water temperature is in agreement with the climate station, indicating that there was no thermal contamination in the proximity of the sampling site. Values of the total alkalinity and hardness indicate that carbonates/bicarbonates and Ca (with a relatively small contribution of Mg) were the dominant ions. Pesticide concentrations were lower than the analytical technique detection limit; therefore, they were not included in Table 1. Of the total of measured variables, low DO, high ammonium, and high Cu levels stand out. The WQI is 7.3, indicating a moderate degree of contamination.

In the bioassay media, the average effective values of cadmium in each exposure treatment (mean ± SEM; n = 4) were < 0.5 *μ*g/L for NC and CP, < 0.5 *μ*g/L for RR, 2450 ± 50 *µ*g/L for RR+Cd, and 2550 ± 50 *µ*g/L for Cd. The range of the daily controlled parameters was DO: 7.1–8.9 mg O_2_/L; pH: 7.4–7.9 U pH; and hardness: 65–100 mg CaCO_3_/L. Only 18% mortality was registered in the Cd treatment.

### 3.2. Gill and Liver Histopathology

**Figure 1:**
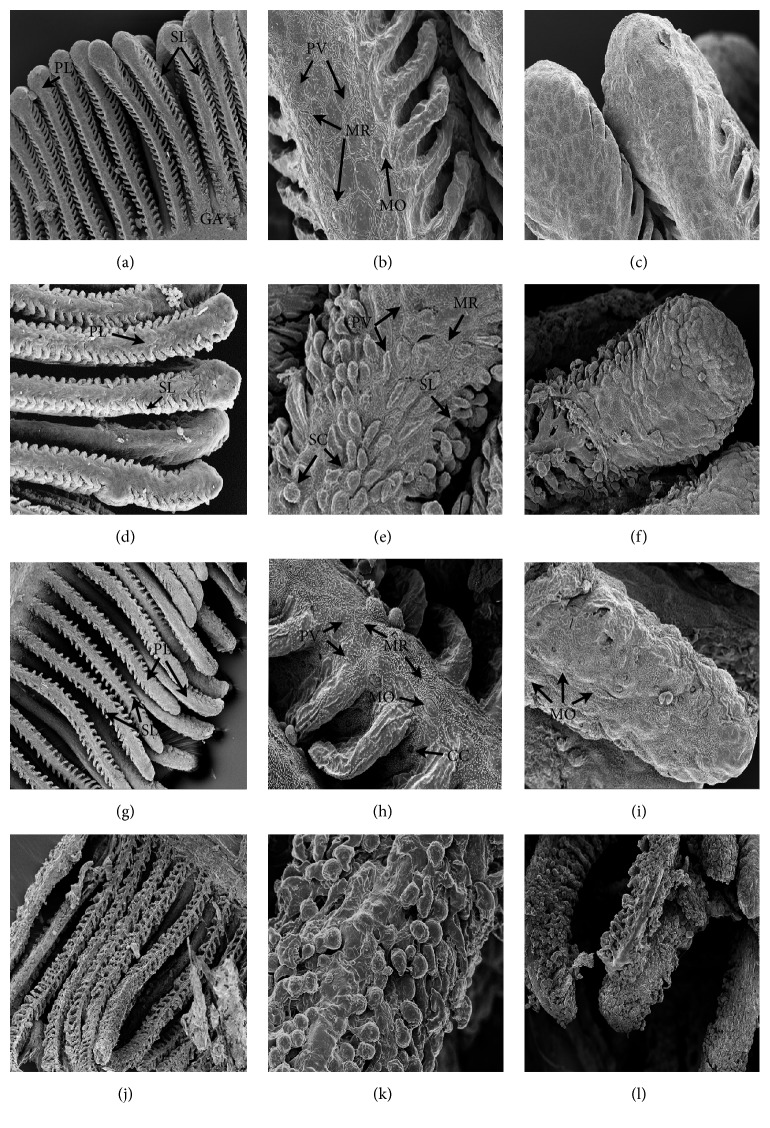
Gill filaments histological sections of* Cnesterodon decemmaculatus* exposed to different treatments for 96 h. Treatments: NC (negative control, MHW); RR (surface water of the Reconquista River); RR+Cd (surface water of the Reconquista River with 2 mg Cd/L); Cd (2 mg Cd/L). Photomicrographs were taken under a Philips XL series 30 or a Carl Zeiss NTS SUPRA 40 scanning electron microscope. Slides (a), (b), and (c) correspond to fish exposed to NC: (a) normal arrangement of primary lamellae (PL) and secondary lamellae (SL) on the gill arc (GA) (350 X); (b) enlarged portion of (a): pavement cell (PV), concentric microridge (MR); mucous cell (MO) (3500X); (c) apical portion of a PL (2000X). Slides (d), (e), and (f) correspond to fish exposed to Cd: (d) primary (PL) and secondary lamellae (SL) (200 X); (e) aspect of a portion of PL with its SL, evident heeling (SC), and disorganization of the epithelium (3500X); (f) apical portion of a PL, showing epithelium disorganization, lifting and swelling of the pavement cells (2000X). Slides (g), (h), and (i) correspond to fish exposed to RR: (g) primary (PL) and secondary lamellae (SL) (350 X); (h) enlarged portion of a PL showing pavement cell (PV), concentric microridge (MR), mucous cell (MO), and chloride cell (CC) (4000X); (i) apical portion of a PL showing edema and abundant mucous cells (MO) flanked by pavement cells (3000X). Slides (j), (k), and (l) correspond to fish exposed to RR+Cd: (j) primary (PL) and secondary lamellae (SL) (500 X), (k) enlarged portion of a PL showing marked epithelium disorganization (5000X); (l) apical portion of a PL showing different degrees of epithelium disorganization, varying from edema to complete disorganization of epithelial architecture (1000X).

Scanning electron photomicrographs of the gills of* C. decemmaculatus* reveal remarkable structural differences between the NC and treated individuals (Figure 1). In the NC (Figures 1(a)–1(c)), gills exhibited a normal arrangement of primary and secondary lamellae. The primary lamellae or gill filaments are oriented perpendicular to and along the gill arch (GA), while the secondary lamellae resemble thin semi-circular “lapels” arranged in bilateral symmetry with respect to the former. The secondary lamellae of* C. decemmaculatus *are relatively short compared to those of another teleost [33]. The pavement cells, which are the dominant cell type, have concentric microridges (MR) on their surface and are surrounded by tight junctions. Some mucous cells (MO)—less exposed to the surface—are scattered in the epithelium. The gills of Cd-treated fish show epithelial disorganization, edema in the primary and secondary lamellae, and fusion of adjacent lamellae (Figures 1(d)–1(f)). Epithelial disorganization involves lifting, swelling, and shedding of the cells, which become spherical in shape especially at the tip of primary lamellae. The gills of fish exposed to RR samples show more labile intercellular junctions, a larger number of chloride cells (CC), and mucous cells (MO) and the presence of aneurysms (AN) in vessels of the secondary lamellae in comparison to the control fish (Figures 1(g)–1(i)). These pathological changes are more severe in the gills of fish exposed to the RR+Cd treatment; this treatment also caused important desquamation of the epithelium together with fewer microvilli on the surface of pavement cells (Figures 1(j)–1(l)).

**Figure 2:**
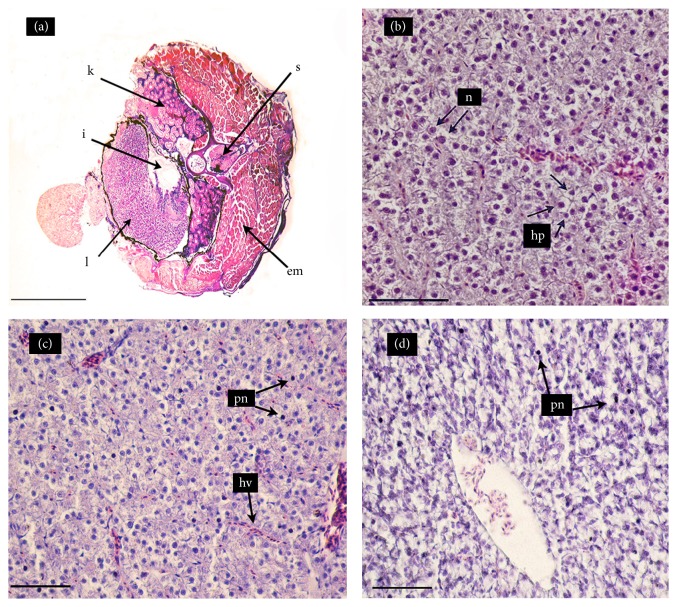
Hematoxylin-eosin stained cross-sections of the mid-section of* Cnesterodon decemmaculatus* exposed to different treatments for 96 h. Treatments: NC (negative control, MHW); RR (surface water of the Reconquista River); RR+Cd (surface water of the Reconquista River with 2 mg Cd/L). (a) Fish exposed to NC (5X): liver (L); intestine (I); kidney (K); epiaxial muscle (EM); and spine (S). (b) Liver of fish exposed to NC (40X): normal hepatocytes (hp) with one nucleus (n) each. (c) Liver of fish exposed to RR (40X): hepatic vein (hv) and hepatocytes with pyknotic nuclei (pn) in the parenchyma and thin sinusoids in the parenchyma. (d) Liver of fish exposed to RR+Cd (40X): marked increase in the number of hepatocytes with pyknotic nuclei (pn).

Figure 2 shows cross-sections of the livers of* C. decemmaculatus* exposed to the NC and to RR with and without a contamination pulse of 2 mg Cd/L.  Table 2 shows the quantification of the histological damage in terms of the number of melanomacrophage centers, hemorrhages, and pyknotic hepatocytes. The liver of* C. decemmaculatus* is a simple unlobed organ without a portal system, as in most teleost. In the NC, the liver (L) is found in a ventrolateral position, partially surrounding the intestine (I) and occupying a large proportion of the abdominal cavity, whereas the kidney (K) is placed below the epiaxial musculature and the vertebral column (Figure 2(a)). When viewed at higher magnification (Figure 2(b)), parenchymal hepatocytes are observed to be arranged in cord-like structures with a normal architecture; hepatocytes present a homogeneous cytoplasm and a large central or subcentral spherical nucleus with concentric nucleoli. The histological analysis of the livers of fish exposed to the RR treatment (Figure 2(c)) revealed hyperemia (congestion) of hepatic veins (hv) and adjacent sinusoids and a significant increase in hepatocytes with pyknotic nuclei (pn), as compared to the control (Table 2). The RR+Cd treatment increased karyopyknosis (Figure 2(d)) by an order of magnitude (Table 2).

### 3.3. Genotoxic Biomarkers

**Figure 3:**
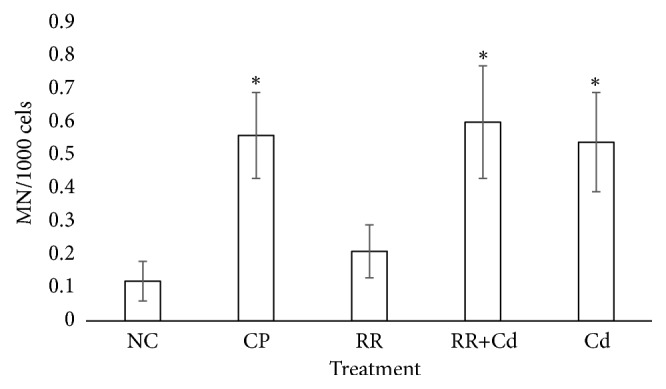
Analysis of micronuclei frequency in peripheral blood erythrocytes of* Cnesterodon decemmaculatus* exposed to different treatments. Treatments: NC (negative control, MHW); CP (positive control, 5 mg cyclophosphamide/L); RR (surface water of the Reconquista River); RR+Cd (surface water of the Reconquista River with 2 mg Cd/L); Cd (2 mg Cd/L). *∗* p < 0.05 compared to NC. Number of individuals analyzed in each treatment: 16-22.

**Figure 4:**
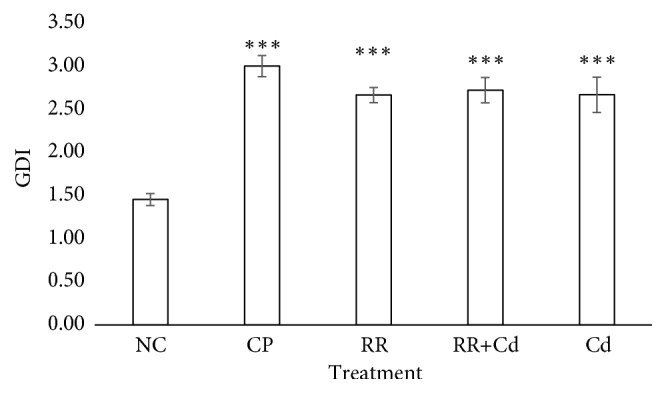
Analysis of DNA damage measured by comet assay in peripheral blood erythrocytes of* Cnesterodon decemmaculatus* exposed to different treatments, as indicated by the genetic damage index (GDI). Treatments: NC (negative control, MHW); CP (positive control, 5 mg cyclophosphamide/L); RR (surface water of the Reconquista River); RR+Cd (surface water of the Reconquista River with 2 mg Cd/L); Cd (2 mg Cd/L). *∗∗∗* Significant differences with respect to control values at p < 0.001. Number of individuals analyzed in each treatment: 8-10.

**Figure 5:**
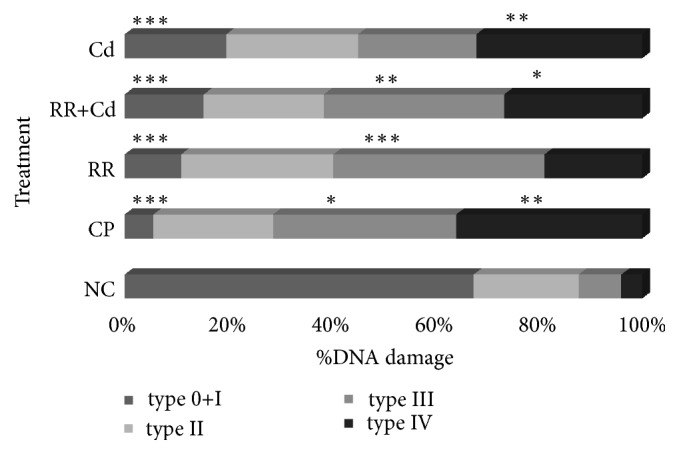
Percentage of DNA damage in peripheral blood erythrocytes of* Cnesterodon decemmaculatus* exposed to different treatments, based on five types of nucleoids (0+I; II; III and IV). Treatments: NC (negative control, MHW); CP (positive control, 5 mg cyclophosphamide/L); RR (surface water of the Reconquista River); RR+Cd (surface water of the Reconquista River with 2 mg Cd/L); Cd (2 mg Cd/L). Significant differences with respect to control values at *∗* p < 0.05, *∗∗* p < 0.01, and *∗∗∗* p < 0.001. Number of individuals analyzed in each treatment: 8-10.

The results of the MN assays are presented in Figure 3, and the results of the comet assay are shown in Figures 4 and 5. The frequency of MN increased significantly with respect to the NC in the CP, RR+Cd, and Cd treatments. The GDI increased significantly in all treatments compared to the NC (p < 0.001). The statistical analysis indicated that the increase in the GDI was due to an increase in the frequency of type II-IV comets (p < 0.05) and a concomitant decrease in the frequency of type 0-I comets (p < 0.05). The percentage of nondamaged erythrocytes in the NC was about 70% whereas it decreased to 10 to 20% in fish exposed to the CP, Cd, RR+Cd, and RR treatments.

### 3.4. Condition Factor and Biochemical Biomarkers

**Figure 6:**
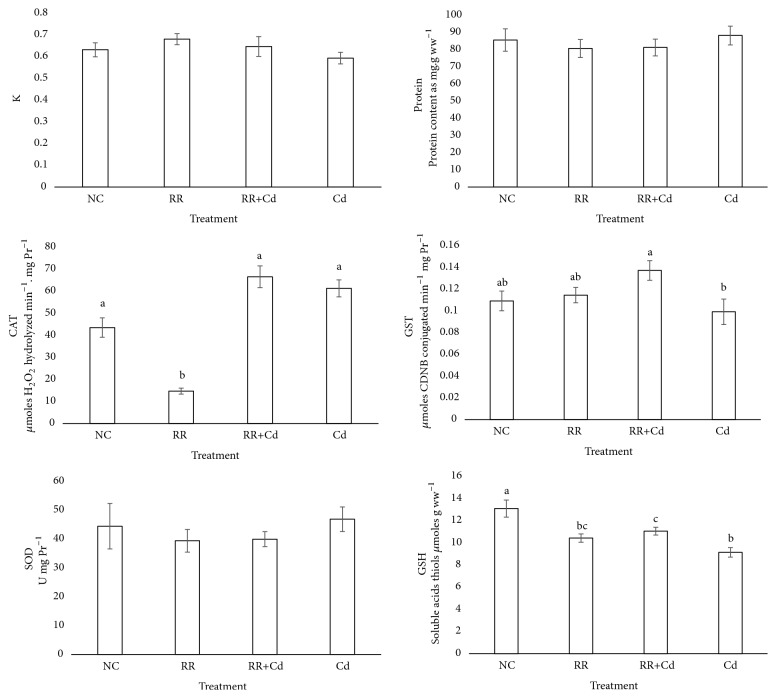
Biomarkers and morphological parameters of oxidative stress in* Cnesterodon decemmaculatus* exposed to different treatments for 96 hs. Treatments: NC (negative control, MHW); RR (surface water of the Reconquista River); RR+Cd (surface water of the Reconquista River with 2 mg Cd/L); Cd (2 mg Cd/L). Values are expressed as mean ± SEM. Treatments not sharing a common letter are statistically different from each other (ANOVA followed by Tuckey test, p < 0.05). Number of individuals analyzed in each treatment: 16-22.

The values of the condition factor and biochemical biomarkers obtained from body midsections are shown in Figure 6. No significant differences in Fulton's condition factor (K), SOD activity, and protein content were found among all treatments.

Fish exposed to the RR treatment showed a significant decrease in CAT with respect to the NC, RR+Cd and Cd treatments. Significant differences in GST were observed between the RR+Cd and Cd treatments. Compared to the NC, a statistically significant decrease in GSH was observed (p < 0.05) in the RR, RR+Cd, and Cd treatments, with the latter showing the largest decrease.

## 4. Discussion

Fish are largely being used for the assessment of the quality of aquatic environment and as such can serve as bioindicators of environmental pollution. Our results show that the use of set of biomarkers could contribute to establish which sensitive endpoint indicators would be better to detect pollution in contaminated waters.

The geographical distribution of* C. decemmaculatus* covers the entire Pampasic region, inhabiting a variety of habitats including some extremely polluted areas [7]. In fact,* C. decemmaculatus* has been found in aquatic environments with concentrations of heavy metals and pesticides well above the maximum limits allowed [11, 45]. This species has been proposed as a test species for monitoring bioassays and is in the process of validation, so, it is necessary to increase the knowledge of its responses to different pollutants, experimental conditions, and assessment processes. We investigated the effect of receiving waters at the headwaters of the Reconquista River with and without a contaminant pulse of Cd on different biomarkers in adult* C. decemmaculatus*, including a positive control for Cd.

The analysis of the samples used in this study indicated a moderate contamination level based on the WQI value (Table 1), copper and ammonium concentrations above the recommended levels for the protection of aquatic biota, and a low DO concentration. Our results showed that in our experimental conditions the water quality of the headwaters of the Reconquista River did not affect fish survival. Other studies conducted in the area using fish and amphibians have reported similar results [11, 46–48]. In spite of the nonlethal effect, several of the biomarkers tested have been significantly modified in regard to the NC, especially the branchial morphology, hepatic parenchyma, and DNA damage.

It is known that fish gills are involved in many important functions such as respiration, osmoregulation, and excretion. They are particularly sensitive to changes in the quality of the water because their large surface area is in close contact with the external environment and because the respiratory exchange takes place through a thin epithelium. Hence, they are the primary target organs for pollution [49]. Regarding fish respiratory physiology, changes in gill tissue are characterized by an increased diffusion distance for oxygen (water–blood) and a smaller respiratory surface, which may lead to functional hypoxia [50, 51]. In our study, SEM analysis of Cd-exposed fish gills showed epithelial disorganization, mainly desquamation of the epithelium together with a decreased number of microvilli on the surface of pavement cells, and fusion of adjacent secondary lamellae. Comparable results were found in another teleost exposed to Cd [33]. The gill epithelium of RR-exposed fish exhibited a higher abundance of chloride and mucous cells. Chloride cell proliferation has been observed in fish exposed to heavy metals, which can be interpreted as a compensation response for ion loss or as a mechanism to increase the excretion rate of toxicants [52]. Secondary lamellae also suffered vascular lesions such as aneurysms, which result from the disruption of pillar cells and cause hemorrhage. These results suggest a decreased respiratory capacity and the occurrence of osmotic imbalance due to toxicant stress. Lamellar fusion, as a result of the excessive proliferation of epithelial cells of the filament, shown in Cd-treated fish gills is a natural defense mechanism to protect the lamellar epithelium from direct contact with toxic agents [53].

The liver has been recognized as a target organ for different pollutants. In our study, the hepatic histopathological analysis revealed hyperemia and congestion of blood vessels, which might be associated with circulatory disturbances related to pathological conditions of blood and tissue fluid flow and regressive changes [54]. Interestingly, we found karyopyknosis in fish exposed to the water of the Reconquista River, either with or without Cd addition. Although the RR+Cd treatment induced a larger number of pyknotic nuclei, the hepatic function of fish inhabiting the Reconquista River may be impaired. Karyopyknosis, which is the irreversible condensation of chromatin in the nucleus of a cell undergoing necrosis or apoptosis, is most likely due to the deposition of lipids and glycogen in the hepatocytes [55]. Comparable results have been reported for the liver of* Carassius auratus *exposed to Cr [55] and for* C. decemmaculatus* chronically exposed to chlorpyrifos [56] or 17*α*-ethinylestradiol [57].

The MN test detects irreparable lesions that manifest as chromosome aberrations and/or aneugenic effects [58]. Previous studies have demonstrated the genotoxic effect of Cd on fish [59, 60]. RR treatment did not affect MN frequency, but a significant increase in the MN frequency in the erythrocytes of fish exposed to the Cd and RR+Cd treatments was observed. These results confirm others reported previously with the same experimental conditions [11] and reinforce the role of Cd as a toxic referent for this species. These findings are in concordance with previous studies in which Cd was evaluated. In an early study, we found that concentrations of 0.5 mg Cd/L increased the frequency of MN in juveniles of the common carp* Cyprinus carpio* [61]. Also, acute toxicity of Cd has been reported in root tip cells of* Allium cepa* by increasing the frequency of MN [62], and Cd exposure increased the frequency of MN in polychromatic erythrocytes in rats [63] as well as in the laboratory model zebrafish* Danio rerio* exposed to environmentally relevant concentrations of Cd [64]. Our results are in accord with these observations highlighting the genotoxic potential of Cd as a toxic element for living organisms.

The comet assay is a rapid, simple, and sensitive test that can detect primary DNA lesions and repair in any eukaryotic cell type after xenobiotic exposition. In the present study, we observed a significant increase in the frequency of single strand breaks introduced into DNA as well as an increase in the frequency of GDI in the blood cells of fish exposed to the RR, RR+Cd, and Cd treatments. It is well known that oxidative damage occurs when an excessive generation of ROS occurs, which finally attacks subcellular components, including the DNA molecule [65]. In the present study, we found that all river water treatments have high genotoxicity, revealed by the comet methodology, leading to the generation of single strand breaks. The genotoxicity found could possibly be due to the increased bioavailability of several toxic compounds which can further induce intracellular generation of ROS, revealed by an inhibition of CAT as well as by an increase in the GST levels, and consequently generate high levels of cellular oxidative damage [65].

Antioxidant enzymes and nonenzymatic systems are essential for the conversion of ROS into harmless metabolites and to protect and restore normal metabolism and cell function. CAT and GST are among the most-used antioxidant and biotransformation enzymes, respectively. We observed that CAT activity decreased in animals exposed to the RR treatment, whereas GST activity increased in those exposed to the RR+Cd treatment. In the Cd treatment, neither CAT nor GST response differed from the NC, as previously reported [11]. CAT and GST activities can be increased or inhibited under chemical stress conditions, with responses depending on the intensity and duration of exposure as well as on the species susceptibility [66]. For example, CAT inhibition has been found in the livers of* Rhamdia quelen* exposed to glyphosate [67] and in* C. carpio* exposed to different concentrations of atrazine and chlorpyrifos [68]. Scarcia et al. [69] studied the impact of exposure to contaminated sites in a Pampean river on juveniles of* C. carpio* and* Pimelodella laticeps* and found a significant increase in GST, though CAT remained unchanged.

Fulton's condition factor showed no differences among the treatment groups; values ranged from 0.6 to 0.7, which are close to those reported by other authors [11, 15].

A decrease in GSH in the RR, RR+Cd, and Cd treatments was observed. The observed GSH decrease is probably an indicator of its exhaustion in phase II biotransformation as confirmed by increased GST activity [70]. River water is a complex matrix and the addition of a Cd pulse does not necessarily lead to increased oxidative stress for individuals exposed to this medium.

## 5. Conclusions

Under experimental lab conditions, adults of* C. decemmaculatus* exposed for 96 h to receiving waters from its distribution area presented alterations in markers of genotoxicity, tissue injury of target organs, and oxidative damage. These quantifiable early effects could have a negative impact on the resistance of fish to environmental stressors and their susceptibility to diseases, potentially reducing their capacity to respond to environmental change and, ultimately, their survival. Interestingly, our results suggest that the population of* C. decemmaculatus* living behind the Roggero dam, at the headwaters of the Reconquista River, is most likely under conditions of chemical stress, despite the fact that this reservoir is considered as only slightly polluted based on the WQI and physicochemical parameters. According to our results, for short term exposure, the SEM analysis of gills and histopathological analysis of the liver could be useful tools for target organ damage evaluation as well as the comet assay for DNA damage. We propose, for the biomonitoring of water bodies in the Pampasic region here described, a 96-h laboratory assay protocol using* C. decemmaculatus* as the test organism model. Similarly, microscope analyses of gills and livers as well as the comet methodology in fish could be considered as sensitive endpoint indicators for detecting pollution in contaminated waters.

## Figures and Tables

**Table 1:** Physicochemical parameters and water quality index (WQI) of the water sampled at the Reconquista river.

Parameters	Units	Methodology	Reconquista River water	Argentine Guidelines^a^ (mg/L)
Temperature	°C		11.5	≤ 45
pH	UpH	Hanna field meter	7.9 ± 0.1 (3)	6.5–10
Conductivity	*µ*S/cm	Hach field meter	720 ± 5 (3)	
Turbidity	UNF		94 ± 2 (3)	
Hardness	mg CaCO_3_/L	EDTA titration	65 ± 5 (3)	
Alkalinity	mg CaCO_3_/L	H_2_SO_4_ titration	678.6 ± 5.8 (3)	
Chlorides	mg Cl^−^/L	AgNO_3_ titration	27 ± 1 (3)	
DO	mg O_2_/L	Iodometric	3.4 ± 0.1 (3)	
Ammonium	mg N-NH_4_^+^/L	Colorimetry	0.41 ± 0,03 (3)	0.05–0.47
Nitrites	mg N-NO_2_^−^/L	Colorimetry	0.05 ± 0.01 (3)	≤ 0.06
PRS	mg P-PO_4_^3-^/L	Colorimetry	0.29± 0.004 (3)	
BOD_5_	mg O_2_/L	Iodometric	1.93	≤ 50
COD	mg O_2_/L	Colorimetry	18	≤ 250
COD/BOD_5_			9.33	
WQI			7.33	
Heavy metal	mg/L	Atomic absorption spectrophotometry		
Cr			< 0.005	0.002
Pb			< 0.010	0.001
Cd			< 0.005	0.0001–0.0004
Cu			0.010	0.002–0.004
Zn			0.013	≤ 0.030
As			0.009	≤ 0.03

^a^Argentine Surface Water Guidelines for protection of aquatic life or maximum allowable content in effluents disposed in a water body. Data are expressed as mean ± SEM of three readings.

**Table 2:** Quantification of liver damage: values of the lesions induced by the different treatments. Data are expressed as mean ± SEM. Treatments: NC (negative control, MHW); RR (surface water of the Reconquista River); RR+Cd (surface water of the Reconquista River with 2 mg Cd/L).

Lesions	NC	RR	RR+Cd
Melanomacrophage centers	0.76 ± 0.25 (21)	0.67 ± 0.28 (18)	0.30 ± 0.17 (20)
Hyperemia	3.05 ± 0,47 (21)	2.89 ± 0.40 (18)	3.10 ± 0.64 (20)
Hemorrhages	0.76 ± 0.25 (21)	0.17 ± 0.12 (18)	0.45 ± 0.21 (20)
Hepatocytes with pyknotic nuclei	11.47^**a**^ ± 1.46 (21)	37.22^**b**^ ± 4.14 (18)	123.20^**c**^ ± 7.34 (20)

Number of microscopic fields examined is given in parentheses. Different letters indicate significant differences among treatments (p < 0.05).

Number of individuals analyzed in each treatment: 17-20.

## Data Availability

The data used to support the findings of this study are available from the corresponding author upon request.
